# Interactive effects between plant functional types and soil factors on tundra species diversity and community composition

**DOI:** 10.1002/ece3.2548

**Published:** 2016-10-17

**Authors:** Maitane Iturrate‐Garcia, Michael J. O'Brien, Olga Khitun, Samuel Abiven, Pascal A. Niklaus, Gabriela Schaepman‐Strub

**Affiliations:** ^1^ Department of Evolutionary Biology and Environmental StudiesUniversity of ZurichZurichSwitzerland; ^2^ Estación Experimental de Zonas ÁridasConsejo Superior de Investigaciones CientíficasAlmeríaSpain; ^3^ Komarov Botanical InstituteRussian Academy of ScienceSt. PetersburgRussia; ^4^ Department of GeographyUniversity of ZurichZurichSwitzerland

**Keywords:** active layer thickness, carbon‐to‐nitrogen ratio, cellulose‐to‐lignin ratio, edaphic factors, moisture, nonvascular species, pH, vascular species, vegetation cover

## Abstract

Plant communities are coupled with abiotic factors, as species diversity and community composition both respond to and influence climate and soil characteristics. Interactions between vegetation and abiotic factors depend on plant functional types (PFT) as different growth forms will have differential responses to and effects on site characteristics. However, despite the importance of different PFT for community assembly and ecosystem functioning, research has mainly focused on vascular plants. Here, we established a set of observational plots in two contrasting habitats in northeastern Siberia in order to assess the relationship between species diversity and community composition with soil variables, as well as the relationship between vegetation cover and species diversity for two PFT (nonvascular and vascular). We found that nonvascular species diversity decreased with soil acidity and moisture and, to a lesser extent, with soil temperature and active layer thickness. In contrast, no such correlation was found for vascular species diversity. Differences in community composition were found mainly along soil acidity and moisture gradients. However, the proportion of variation in composition explained by the measured soil variables was much lower for nonvascular than for vascular species when considering the PFT separately. We also found different relationships between vegetation cover and species diversity according the PFT and habitat. In support of niche differentiation theory, species diversity and community composition were related to edaphic factors. The distinct relationships found for nonvascular and vascular species suggest the importance of considering multiple PFT when assessing species diversity and composition and their interaction with edaphic factors. *Synthesis*: Identifying vegetation responses to edaphic factors is a first step toward a better understanding of vegetation–soil feedbacks under climate change. Our results suggest that incorporating differential responses of PFT is important for predicting vegetation shifts, primary productivity, and in turn, ecosystem functioning in a changing climate.

## 1 Introduction

Niche theory predicts that vegetation communities are coupled with abiotic factors because species both respond to and influence local climate and soil characteristics (Medinski, Mills, Esler, Schmiedel, & Jürgens, 2010; van der Putten et al., 2013; Valladares, Bastias, Godoy, Granda, & Escudero, 2015; Wookey et al., 2009). Changes in vegetation communities might thus affect climate through vegetation–radiation and vegetation–soil feedbacks among others (Beringer, Chapin, Thompson, & McGuire, 2005; Chapin et al., 2000). These interactions may be especially important in high‐latitude ecosystems which are expected to undergo large shifts in vegetation distribution as climate changes (Jägerbrand, Lindblad, Björk, Alatalo, & Molau, 2006). Harsh environmental conditions, poorly developed soils, and active cryogenic processes characterize high‐latitude ecosystems, providing a mosaic of vegetation communities across the landscape (Billings & Mooney, 1968; Chernov & Matveyeva, 1997; Walker, 2000). In these ecosystems, community assembly is mainly affected by edaphic factors and biotic interactions between vascular and nonvascular species (Cornelissen et al., 2001; Doxford, Ooi, & Freckleton, 2013; Gornall, Woodin, Jónsdóttir, & van der Wal, 2011; Jägerbrand et al., 2006). However, research has largely ignored the role of plant functional types (PFT) other than vascular plants, despite the differential processes interacting with PFT to promote community assembly and ecosystem functioning (Madrigal‐González, García‐Rodríguez, & Alarcos‐Izquierdo, 2012).

PFT are groupings of species which respond similarly to environmental conditions and affect ecosystem processes in similar ways (Lavorel, McIntyre, Landsberg, & Forbes, 1997). PFT have been broadly used in climatic models which predict vegetation shifts (Walker, 2000). However, PFT classification and its level of detail depend on the spatial scale and the ecosystems and ecological processes under research. In Arctic research, tundra vegetation is divided, in a first step, into vascular and nonvascular PFT (Chapin, Bret‐Harte, Hobbie, & Zhong, 1996; Walker, 2000). The vascular PFT includes shrubs and herbaceous (graminoids and forbs), while the nonvascular PFT comprises bryophytes and lichens (cryptogams). Both functional types are expected to change their distribution under future climatic conditions (Cornelissen et al., 2001; Myers‐Smith et al., 2011). Although the nonvascular functional type may respond differently to the environmental changes, frequently only several levels of vascular functional types are used in global vegetation models. The omission of nonvascular PFT could result in inaccurate predictions of tundra ecosystem responses to climate change due to the higher abundance of nonvascular than vascular species in high‐latitude ecosystems and their strongly different responses to environmental factors (Chapin & Shaver, 1996; Matveyeva & Chernov, 2000; Tenhunen, Lange, Hahn, Siegwolf, & Oberbauer, 1992).

Community assembly is driven by biotic interactions, but also depends on environmental conditions (Cornwell & Ackerly, 2009). Soil characteristics are strong predictors of species diversity and composition, especially in heterogeneous environments where the spatial distribution of vegetation species depends on their niche preferences (Björk et al., 2007; Sundqvist et al., 2011; Valladares et al., 2015). For example, studies show a strong correlation between soil acidity or moisture and species diversity (Chytrý et al., 2007; Gough, Shaver, Carroll, Royer, & Laundre, 2000). Diversity and distribution of species are also associated with patterns of mineral nitrogen and phosphorus availability in the soil, which is particularly important in ecosystems with low soil nutrient availability such as tundra (Arnesen, Beck, & Engelskjøn, 2007; Gough & Hobbie, 2003; Wardle, Gundale, Jäderlund, & Nilsson, 2013). Climate change may have important effects on soil characteristics through increased soil temperature, fluctuations in moisture and enhanced nutrients (IPCC 2013; Keller, White, Bridgham, & Pastor, 2004; Seneviratne et al., 2010). As a consequence, species composition and diversity may change, likely affecting important ecosystem functions such as primary productivity (Balvanera et al., 2006; Cardinale et al., 2011; Hector et al., 1999).

Arctic tundra vegetation is adapted to harsh environmental conditions, such as extremely low temperatures, precipitation, and soil nutrient availability (Billings & Mooney, 1968). The vegetation grows slowly due to short growing seasons (<3 months) and is covered by snow for the rest of the year. The dependence of vegetation on edaphic factors, combined with small‐scale heterogeneity in soil characteristics, promotes the patchy distribution of communities in tundra (Lantz, Gergel, & Kokelj, 2010; Mod, Le Roux, & Luoto, 2014; Walker, 2000). Furthermore, nonvascular species can change the diversity and composition of vascular plant communities in tundra due to their strong effects on soil characteristics and on germination and establishment of seedlings (Doxford et al., 2013; Gornall et al., 2011; Sedia & Ehrenfeld, 2003). Despite the importance of cryptogams, most studies on the relation between edaphic factors and species diversity and community composition have focused on vascular plants (Heikkinen & Neuvonen, 1997; Gough et al., 2000; Sundqvist et al., 2011; see exceptions: Jägerbrand et al., 2006; Löbel, Dengler, & Hobohm, 2006; Lang et al., 2012).

In this study, we investigated the relation between vegetation and soil variables in a tundra ecosystem in northeastern Siberia in order to better understand the interactions between species diversity, community composition, and soil variables, as well as the underlying edaphic factors promoting niche differentiation. We hypothesized that (1) species diversity and community composition of nonvascular and vascular PFT are related to soil variables; (2) vegetation cover correlates positively with species diversity; and (3) the relationships among species diversity, community composition, vegetation cover, and soil variables are PFT‐specific. To test our hypotheses, we assessed the vegetation species diversity, community composition, cover, and several soil variables at two locations differing in topography and soil characteristics: a Pleistocene river terrace and a drained thaw lake basin. Due to their importance for tundra ecosystem functioning and vegetation–climate feedbacks, nonvascular species were considered in this study.

## 2 Materials and Methods

### 2.1 Study area and sampling design

The study area is located in the Kytalyk nature reserve in the Yana‐Indigirka Lowlands, Yakutia, northeastern Siberia (70°49′N, 147°28′E, 10 m.a.s.l.), in the continuous permafrost Arctic region. The mean annual air temperature is −13.1°C with minimum and maximum monthly means of −33.5°C in January and 11.3°C in July, and the mean annual precipitation is 232 mm (1981–2013, WMO station 21946, Chokurdakh), mainly occurring during the growing season (about mid‐June to end‐August). Although 2013 was a slightly cold and dry year, both years of the study (2013 and 2014) had similar climates to the long‐term averages. Three geomorphological units are present at the study area: a Pleistocene river terrace, a drained thaw lake basin—hereinafter referred to as ridge and lakebed—and a flood plain (Blok et al., 2010). Two cryogenic relief forms are typical on the lakebed: low‐ and high‐centered polygonal complexes. In the high‐centered polygonal complex, wet hollows alternate with elevated polygons, which are higher and better drained.

The main vegetation present in the area is tussock‐sedge tundra with abundant dwarf shrubs on the ridge and, on the lakebed, sedge wetlands in the hollows and dwarf birch–moss communities on the polygons. High willow copses are characteristic for the flood plain. The tussock‐sedge tundra is formed by low vegetation (3–25 cm) and comprises sedges (mainly *Eriophorum vaginatum*), deciduous (*Betula nana, Salix pulchra*, and *Vaccinium uliginosum*) and evergreen dwarf shrubs (*Cassiope tetragona*,* Dryas octopetala*,* Ledum palustre,* and *Vaccinium vitis‐idaea*), mosses (mainly *Aulacomnium* spp., *Dicranum* spp., *Hylocomnium splendes,* and *Tomentypnum nitens*), and lichens (mainly *Cetraria islandica, Flavocetraria cucculata*, and *Peltigera* spp.). The sedge wetland (20–40‐cm height) is dominated by *Eriophorum angustifolium* and peat mosses (*Sphagnum* spp.,). The dwarf birch–moss communities are commonly dominated by *B. nana* (15–30‐cm height) and various mosses (*Dicranum* spp., *Polytrichum* spp., and *Aulacomnium* spp.,) with rather abundant grasses (*Calamagrostis holmii* and *Arctagrostis latifolia*) and lichens (*C. islandica* and *F. cucculata*). In the transition areas between hollows and polygons, the vegetation is 3–25‐cm height. When the transitional area is closer to hollows, the vegetation is dominated by *Sphagnum* spp. with sparser cover of *E. angustifolium* and deciduous dwarf shrubs (*B. nana, S. pulchra,* and *Salix fuscenses*), while the vegetation is made up of evergreen dwarf shrubs (*V. vitis‐idaea*), mosses, and abundant lichens (mainly *F. cucculata*) with spare cover of *B. nana* when the area is closer to polygons (see Table S1, for species name authority).

**Figure 1:**
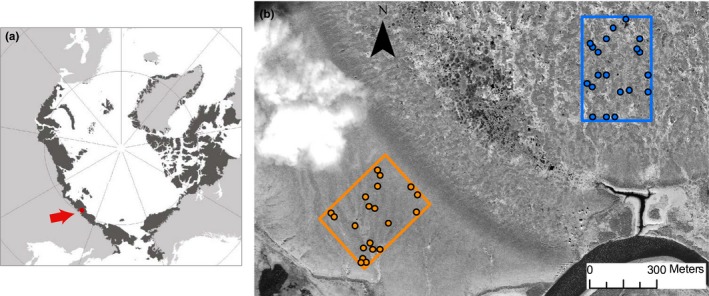
(a) Location of the study area (red point). In dark gray, extent of Arctic tundra (data from Walker et al. 2005); (b) spatial distribution of the 40 plots selected to assess the species diversity: 20 plots placed on the lakebed (blue) and 20 plots on the ridge (orange). The background of the figure is a GeoEye‐1 satellite image from August 2010

We selected two contrasting habitats for the sampling: lakebed and ridge, to ensure that the main vegetation types present in the study area were included. We established 40 observational plots of 0.50 × 0.50 m in an area of 300 × 400 m on the ridge and lakebed (20 plots each). Thirty plots were established at the beginning of the 2013 growing season at random locations and, to improve replication for some of the communities, 10 additional plots were added at the beginning of the growing season of 2014 (Figure 1).

### 2.2 Species diversity, community composition, and vegetation cover

In order to assess the species diversity and community composition, we used a plot‐size grid divided into 25 quadrats of 0.10 × 0.10 m. We placed the grid on each of the 40 selected plots and identified all the species, including cryptogams, within each quadrat. The diversity surveys were carried out during the mid‐growing season in 2013 (6–10th July on the plots selected in 2013) and 2014 (7–8th July on the plots selected in 2014). Vegetation samples were collected when field identification was difficult, which was the case for practically all bryophytes and lichens, and sent to the Komarov Botanical Institute (Russian Academy of Sciences) for identification. We further assigned each species to one of the two following plant functional types (PFT): vascular (shrubs and herbaceous) and nonvascular (bryophytes and lichens) (sensu Walker, 2000). We described the diversity for every plot and PFT using the species richness (number of species of each PFT present in a plot) and Shannon–Wiener diversity index (Shannon, 1948). The community composition was defined as the list of species on each plot including their abundance (estimated as number of grid quadrats where a species was present).

We estimated vegetation cover using a modified point quadrat method (Jonasson, 1988). We placed the grid described above on each of the 30 plots selected in 2013 and passed a metal rod (2‐mm diameter) vertically down at every quadrat intersection. We registered all the contacts with the vegetation, including woody parts and standing litter, from the top layer to the moss layer bottom. The cover was determined adding the number of hits for all the species belonging to each PFT on every plot. The cover surveys were carried out during the mid‐growing season in 2013 (20–30th July).

### 2.3 In situ measurements of soil variables

We measured soil temperature (ama‐digit ad15th digital thermometer, Amarell GmbH & Co., Germany), soil acidity (DM‐13 pH‐meter, Takemura Electric Works, Ltd., Japan), and soil moisture (Theta Probe ML1, Delta T Devices Ltd., UK) in five quadrats of the grid used for the diversity assessment at 10‐cm depth in every plot. We also measured active layer thickness as the distance between permafrost and soil surface, including the moss layer if present. For that, we introduced a metal rod with centimeter scale vertically into the soil to the depth of resistance at the sampling locations where the other soil variables were measured. For every soil variable and plot, the five quadrat measurements were averaged. We measured the soil variables in mid‐July 2014 in all 40 plots. Due to the slow turnover in species composition of tundra communities, we assume that between‐plot differences are more important in explaining species diversity and composition compared to variability between two consecutive years.

### 2.4 Soil sampling and analysis

We sampled two soil cores per plot (4.8 cm diameter × 5.3 cm height) in mid‐July 2013 (30 plots) and 2014 (10 plots added this year). We determined bulk density by air‐drying the soil samples for 3 weeks. Once in the laboratory, the samples were oven‐dried at 70°C for 48 hr, ground, sieved through a 2‐mm mesh, and milled. Carbon and nitrogen content were analyzed in subsamples of about 2.5–3.0 mg using a TruSpec Micro CHN analyser (Leco Corporation, USA). Then, the ratio carbon‐to‐nitrogen was calculated. We determined the cellulose and lignin content in milled 10 mg subsamples by diffuse reflectance infrared Fourier transform spectroscopy (Tensor 27, Bruker Optics GmbH, Fällanden, Switzerland). Spectra were acquired by averaging 64 scans per sample at 4 cm^−1^ resolution over the range 4,000–400 cm^−1^. Powdered KBr was used to create a reference background spectrum and chernozemic soils (Hildesheim‐Braunschweig, Germany) as standard material for the calibration curve. We integrated the peaks corresponding to cellulose (1,260–1,210 cm^−1^) and lignin (1,510–1,500 cm^−1^) and calculated the cellulose‐to‐lignin ratio. Every soil variable determined was averaged per plot.

### 2.5 Data analysis

We analyzed species diversity as a function of site, PFT, and their interaction, with a linear mixed‐effect model. The fixed terms were site (factor with two levels: ridge and lakebed) and PFT (factor with two levels: vascular and nonvascular). Plot was fitted as random factor (40 levels).

In order to explore the relationships between species diversity and soil variables across the 40 plots, standardized soil data were subjected to a principal component analysis (PCA) using the vegan package version 2.3‐1 (Oksanen, 2015) in R (http://r-project.org). Then, species diversity was analyzed as a function of the interactions of site and PFT with the loadings of the two‐first PC axes (fixed terms) using a linear mixed‐effect model. Plot was considered a random term (40 levels).

To test the relationship between community composition and soil variables, the species abundances were subjected to a canonical correspondence analysis (CCA) using the vegan package. The species data were scaled to unit variance to account for differences in abundance distribution (rare species). We constrained the ordination using the standardized soil variables.

We used a linear mixed‐effect model to test whether vegetation cover was sparser in communities with lower species diversity than in more diverse communities. We analyzed the cover and diversity data that were collected in 2013. We considered the interaction among species diversity (a continuous variable), PFT (two factors: vascular and nonvascular) and site (two factors: ridge and lakebed) a fixed term and plot as a random term (29 levels; to facilitate the analysis, one lakebed plot corresponding to a community without nonvascular species was eliminated). Vegetation cover was expressed as number of hits per grid quadrat. Two extreme values of the vascular cover on the ridge were detected based on model residual values that were more than three times and a half the median absolute deviation (Pascal package). The extreme values were removed prior to analyzing the relation between cover and Shannon–Wiener index.

We performed the statistical analysis using R 3.2.2. The linear mixed‐effect models we used to test our hypotheses were fitted in ASReml (ASReml 3.0, VSN International Ltd., UK).

## 3 Results

### 3.1 Species diversity

Twenty‐five vascular and 53 nonvascular species (29 lichen and 24 bryophyte species) were identified in the 40 plots. Species richness ranged from 1 to 30 species per plot with a mean of 15 species (60% of them were nonvascular). Nonvascular diversity (Fig. S1) was significantly higher than vascular diversity in both sites (richness *F*_1,39_ = 32.8, *p *<* *.001; Shannon–Wiener index *F*_1,39_ = 28.0, *p* < .001). Species diversity was also significantly higher on the ridge than on the lakebed (richness *F*_1,39_ = 21.1, *p *<* *.001; Shannon–Wiener index *F*_1,39_ = 8.41, *p *<* *.01).

### 3.2 Soil variables

Overall, the soil was acidic (pH < 6.5), rich in organic matter with a low bulk density (mean ± standard deviation: 0.455 ± 0.263 g/cm^3^). Soil organic matter had not decomposed extensively as the cellulose‐to‐lignin and carbon‐to‐nitrogen ratios indicated (1.83 ± 1.00 and 21.7 ± 4.0). The measured soil variables were highly variable among plots and differed between lakebed and ridge, except for soil moisture and carbon‐to‐nitrogen ratio. On average, bulk density, pH, and cellulose‐to‐lignin ratio were lower for soils on the lakebed than on the ridge (Table 1). The active layer was also thinner for lakebed soils, while temperature and carbon and nitrogen contents were higher than for ridge soils. The quality of soil organic matter was comparable between both sites (no differences in the carbon‐to‐nitrogen ratio), although the decomposition rate may have been higher on the lakebed (lower cellulose‐to‐lignin ratio).

**Table 1:** Summary of the soil variables measured on lakebed and ridge plots. Minimum (Min), maximum (Max), mean, and standard deviation (*SD*) values are included

	Lakebed				Ridge			
	Min	Max	Mean	*SD*	Min	Max	Mean	*SD*
Dry bulk density (g/cm^3^)	0.128	0.530	0.310	0.090	0.297	1.20	0.601	0.299
Moisture (%)	17.8	70.2	35.9	19.1	15.3	69.7	38.2	15.3
pH	4.90	6.40	5.84	0.44	5.30	6.60	6.11	0.35
Active layer thickness (cm)	14.1	42.5	24.9	8.8	14.9	47.9	34.6	7.7
Temperature (°C)	3.90	7.90	6.44	1.04	2.50	9.20	4.77	1.40
Carbon content (%)	13.4	42.3	27.2	7.9	2.3	31.7	14.8	9.4
Nitrogen content (%)	0.69	1.95	1.28	0.33	0.13	1.62	0.68	0.44
Carbon‐to‐nitrogen ratio	18.1	35.1	21.4	4.8	18.0	32.5	22.0	3.2
Cellulose‐to‐lignin ratio	0.53	3.30	1.25	0.77	0.76	3.77	2.41	0.86

**Figure 2:**
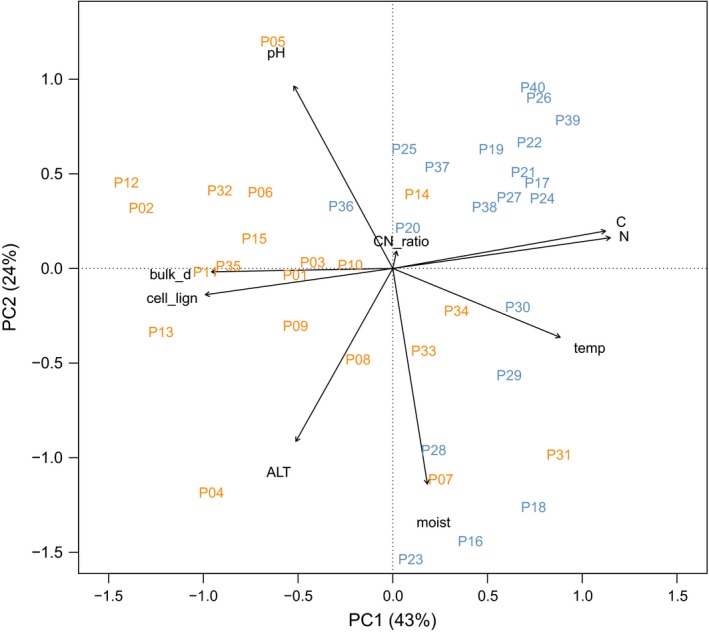
Principal component biplot of the soil variables measured across the 20 plots selected on the ridge (in orange) and the 20 plots selected on the lakebed (in blue). The soil variables include pH, moisture (moist), temperature (temp), active layer thickness (ALT), dry bulk density (bulk_d), carbon content (C), nitrogen content (N), carbon‐to‐nitrogen ratio (CN_ratio), and cellulose‐to‐lignin ratio (cell_lign). The first component explains 43% of the total variance, while the second component explains the 24%

When analyzed with a PCA, soil data were separated into lakebed and ridge plots (site) by the first PC axis (Figure 2). The first PC axis was mainly related to nitrogen and carbon content, cellulose‐to‐lignin ratio, and dry bulk density, explaining 43% of the variation among plots. The second PC axis explained 24% of the total variation in soil data and was related to moisture, pH, and active layer thickness (ALT).

### 3.3 Species diversity‐soil variable relations

**Figure 3:**
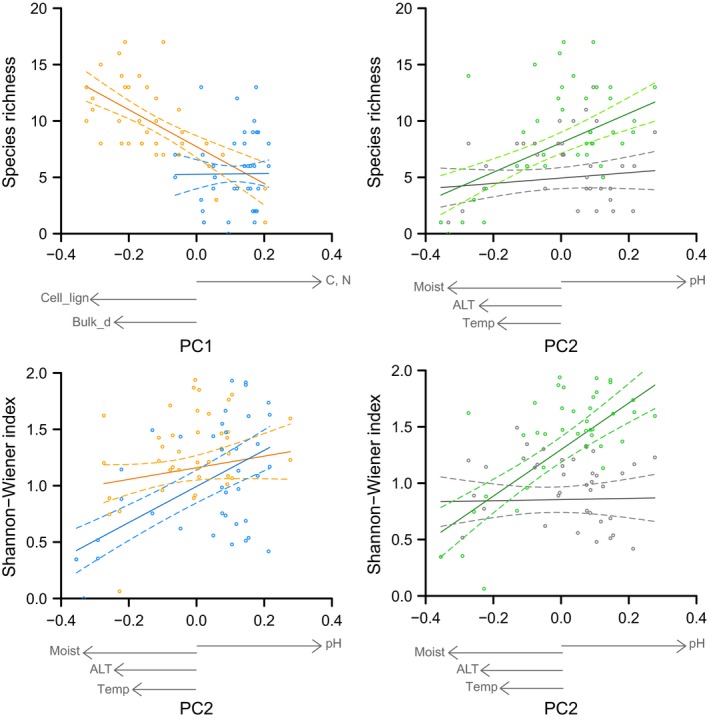
Species diversity relationships with the soil variables by site (lakebed [blue], ridge [orange]), and plant functional type (vascular [gray], nonvascular [green]). The top panels show the species richness and the bottom panels the Shannon–Wiener index relationships. Solid lines are the values predicted by the linear mixed‐effect model, dashed lines are the upper and lower limits of the confidence interval of the predicted values, and points are measured data. The main soil variables comprising the first and second principal component axes (gray arrows) are carbon content (C), nitrogen content (N), cellulose‐to‐lignin ratio (cell_lign), dry bulk density (bulk), pH, moisture (moist), active layer thickness (ALT), and temperature (temp)

To determine the relations between species diversity and soil variables, we used the loadings of the two‐first axes of the PCA. We found that species richness was related to the first PC axis, while Shannon–Wiener index was related to the second PC axis when analyzing the interactive effects of soil variables and site (species richness *F*_1,39_ = 8.16, *p *<* *.01; Shannon–Wiener index *F*_1,39_ = 7.27, *p *<* *.05). These relationships were different between ridge and lakebed. Species richness decreased with increasing carbon and nitrogen content and decreasing cellulose‐to‐lignin ratio and bulk density on the ridge, and remained unresponsive for those variables on the lakebed. The Shannon–Wiener index decreased with soil acidity, moisture, temperature, and ALT on both ridge and lakebed, although the relationship was stronger on the lakebed compared to the ridge (Figure 3).

Species diversity was related to the second PC axis when analyzing the interactive effects of soil variables and plant functional type (PFT) (species richness *F*_1,39_ = 12.5, *p *<* *.01; Shannon–Wiener index *F*_1,39_ = 21.8, *p *<* *.001). This relationship was different for vascular and nonvascular diversity. Nonvascular diversity increased with decreasing soil acidity, moisture, temperature, and ALT, while vascular plants were unresponsive to these variables (Figure 3).

### 3.4 Community composition‐soil variable relations

**Figure 4:**
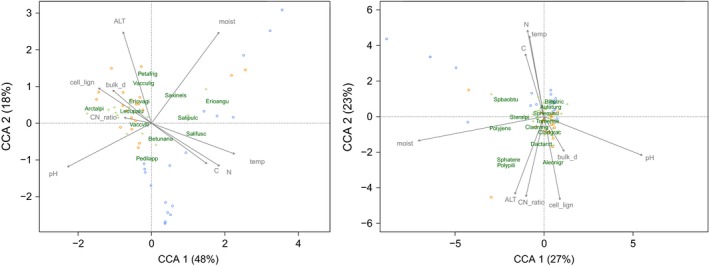
Canonical correspondence analysis (CCA) ordination diagrams of the community compositions for both plant functional types (PFT): vascular (left) and nonvascular (right) on the selected plots constrained by soil variables. The first component explains 48% of the total variance for vascular and 27% for nonvascular PFT, while the second component explains 18% and 23%, respectively. Dominant species (green text), the rest of species (green cross), lakebed plots (blue circles), ridge plots (orange circles), and soil variables (arrows) are shown. See Table S1 for complete name of the species. The soil variables include pH, moisture (moist), temperature (temp), active layer thickness (ALT), dry bulk density (bulk_d), carbon content (C), nitrogen content (N), carbon‐to‐nitrogen ratio (CN_ratio), and cellulose‐to‐lignin ratio (cell_lign)

To explore the relationship between community composition and soil variables, the species abundance was subjected to a canonical correspondence analysis (CCA) constrained by the soil variables. The analysis showed that 37% of the total variance of the community composition was explained by the soil variables when including both PFT together. Species were arranged mainly along the gradients of soil acidity and moisture in this case. The explained proportion of variance of the vascular composition (42%) was higher compared to the nonvascular composition (28%) when considering each PFT alone. Vascular composition was mainly related to the gradients of soil acidity, temperature, moisture, and nitrogen content (principal components of the first CC axis that explained 48% of the response variance) (Figure 4). Nonvascular composition was related to soil moisture and acidity gradients (main components of the first CC axis explaining 27% of the response variance) and, to a lesser extent, gradients of nitrogen content, cellulose‐to‐lignin and carbon‐to‐nitrogen ratios, soil temperature, and ALT (principal components of the second CC axis explaining 23% of the response variance) (Figure 4).

### 3.5 Vegetation cover‐species diversity relations

**Figure 5:**
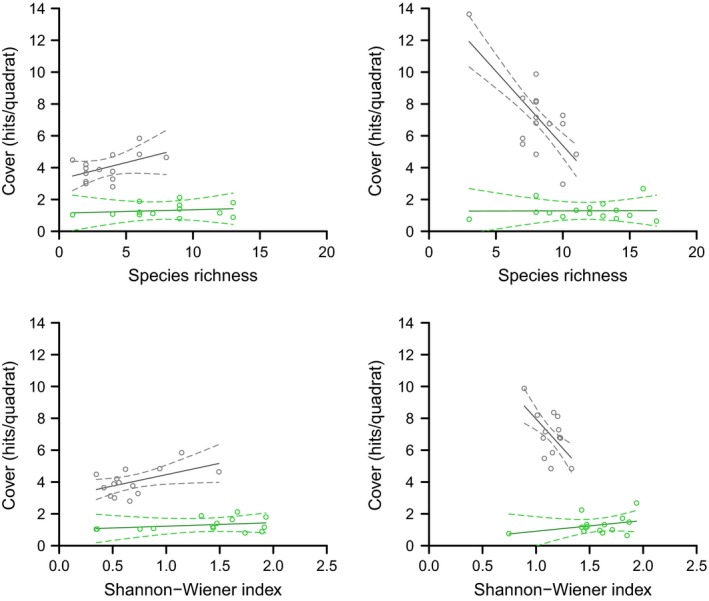
Relationship of the vegetation cover with the diversity. The vegetation cover is expressed as the total number of hits per grid quadrat in each plot for vascular (gray) and nonvascular (green) species. The left panels show the relations found on the lakebed and the right panels on the ridge. Points represent the cover measured in the plots. Solid lines are the predictions of the model and dash lines the confidence interval of the predictions

We found that vegetation cover was related to species diversity (species richness *F*_1,28_ = 19.3, *p *<* *.001; Shannon–Wiener index *F*_1,26_ = 48.5, *p *<* *.001). This relationship was different for site and PFT (Figure 5). Vascular cover was positively related to species diversity on the lakebed. However, the relationship was negative on the ridge. The nonvascular cover increased slightly with species diversity on the lakebed. On the ridge, nonvascular cover increased with the Shannon–Wiener index, but remained constant with the species richness.

## 4 Discussion

We explored the relationships among species diversity, community composition, and soil variables and between vegetation cover and species diversity accounting for nonvascular and vascular plants in an Arctic tundra ecosystem. Our findings show, as we hypothesized, that the relationships were PFT‐specific.

Nonvascular diversity was related to soil variables (negatively related to acidity, moisture, temperature, and ALT), but, surprisingly, vascular diversity was unrelated to soil variables when considering multiple variables simultaneously. Community composition was also related to soil variables, finding important differences in composition along soil acidity and moisture gradients. However, the proportion of variation in composition explained by soil variables was much lower for nonvascular than for vascular species. Because these results show differential responses of PFT to edaphic factors, they confirm the importance of taking into account multiple PFT when studying interactions among species diversity and composition, edaphic factors, and ecosystem functioning, especially in these harsh Arctic ecosystems.

### 4.1 Species diversity

We found low species diversity with higher nonvascular than vascular diversity in the observational plots, which is characteristic of high‐latitude ecosystems (Billings & Mooney, 1968; Gough et al., 2000; Sitch et al., 2007). In these ecosystems, bryophytes and lichens form a predominant PFT that contributes to species diversity due to the fact that cryptogams are often better adapted to harsh environmental conditions than vascular plants (Callaghan et al., 2005; Jägerbrand et al., 2006; Lang et al., 2012; Virtanen et al., 2013).

We also found higher species diversity on the ridge than on the lakebed. Similar to other Arctic areas, the ridge is more wind‐and‐runoff exposed than the lakebed, resulting in a thinner snow layer on the ridge (Bruun et al., 2006; Wahren, Walker, & Bret‐Harte, 2005). As the maximum canopy height in Arctic tundra is conditioned by the thickness of the winter snow layer, communities on the ridge are shorter than on the lakebed (Bilbrough, Welker, & Bowman, 2000; Essery & Pomeroy, 2004). The exclusion of dominant species as a consequence of the exposure, together with less cover shading and leaf litter deposition, may explain the higher species diversity of the ridge communities, especially the greater number of nonvascular species. Furthermore, nano‐relief forms on the ridge resulting from cryogenic processes (tussocks) can provide shelter to several species. In contrast with the ridge, the communities on the lakebed are dominated in abundance and cover by one or two vascular species. On these communities, dominant species prevent other species from establishing and competitively exclude nonvascular vegetation (strong light competition through vascular cover shading and leaf litter deposition), declining the species diversity (Cornelissen et al., 2001; Odland, Reinhardt, & Pedersen, 2015; Sedia & Ehrenfeld, 2003; Startsev, Lieffers, & Landhäusser, 2008; van der Wal, Pearce, & Brooker, 2005).

### 4.2 Soil variables

Similar to other tundra ecosystems, the soil in our study area was acidic and rich in organic matter (Walker, 2000). Low temperature and waterlogging, together with the litter quality and functional composition (e.g., high abundance of evergreen dwarf shrubs) may limit the decomposition of the soil organic matter (Aerts, 2006; Oades, 1988). The characteristic soil heterogeneity of high‐latitude ecosystems was reflected by the high variation of the soil variables among plots (Billings & Mooney, 1968).

Differences found between soil variables on lakebed and ridge might be associated with the vegetation type present in each location. For example, the lower bulk density and soil pH on the lakebed than on the ridge might be related, respectively, to high litter deposition in dwarf birch–moss communities and to methanogenic processes linked to the anaerobic conditions of sedge wetland soils (Christensen, Jonasson, Callaghan, & Havström, 1995; Inglett, Reddy, & Constanje, 2005; Rawls, 1983).

### 4.3 Species diversity‐soil variable relations

We hypothesized that species diversity and edaphic factors were related as reported in several studies (Ma, 2005; Löbel et al., 2006; Gargano, Vecchio, & Bernardo, 2010; Lai et al. 2015). We found that nonvascular diversity was negatively related to soil acidity, moisture, soil temperature, and ALT when considering several soil variables simultaneously. This relationship confirms our hypothesis for cryptogams and is in line with previous findings showing effects of soil variables on species diversity, which in turn can locally modify the soil characteristics (Gornall et al., 2011; Loreau et al., 2001; Sedia & Ehrenfeld, 2003). However, vascular diversity was unrelated to edaphic factors, contrary to what we expected. Although the species composition may vary among locations due to different species physiological tolerance to soil variables (Billings & Mooney, 1968; Eskelinen, Stark, & Männistö, 2009; Theodose & William, 1997), the diversity may remain constant (i.e., different species but same number and abundance), which would overshadow any response. Furthermore, opposite trends in response to individual soil variables (e.g., increase of diversity with pH or decrease with moisture or ALT) might be hidden when considering multiple soil variables at the same time and therefore mask specific relationships.

### 4.4 Community composition‐soil variable relations

Less than half of the variance of the community composition was explained by edaphic factors, suggesting that the rest may be explained, in part, by biotic factors, such as competition (e.g., light shading of nonvascular vegetation by vascular plants) and facilitation (e.g., vascular seedling recruitments and frost protection by nonvascular species) (Gornall et al., 2011; Odland et al., 2015; Sedia & Ehrenfeld, 2003; Virtanen et al., 2013). The presence of a root system and its differences among species might explain the stronger relationship between community composition and edaphic factors for vascular than for nonvascular species.

Nonvascular and vascular PFT comprise species differing in their functional traits (e.g., phenology or rooting system) and, thus, in their niche preferences (Valladares et al., 2015). This niche partitioning might explain our results showing variation in community composition along different soil gradients when accounting for PFT. Vascular community composition was mainly related to soil acidity, temperature, moisture, and nitrogen content. These soil variables can modify the nutrient and water availability, which discriminate among vascular species according to their root characteristics (McKane et al., 2002). Nonvascular composition was related to soil moisture, acidity, temperature, ALT, nitrogen content, and cellulose‐to‐lignin and carbon‐to‐nitrogen ratios. Soil moisture and factors related to soil organic matter (cellulose‐to‐lignin and carbon‐to‐nitrogen ratios) and nutrient availability might discriminate between lichens and bryophytes, as lichens grow in drier and N‐poorer soils (Cornelissen, Lang, Soudzilovskaia, & During, 2007; Sedia & Ehrenfeld, 2003; Virtanen et al., 2013). Soil moisture and acidity might also separate *Sphagnum* spp. from other bryophyte species by their different physiological tolerance (Weston et al., 2015). *Sphagnum* spp. are more abundant in wet acidic soils than other bryophyte species due to their higher tolerance to water stress and soil acidity (Elumeeva, Soudzilovskaia, During, & Cornelissen, 2011; Gough et al., 2000). The relationship between nonvascular composition and ALT might be associated with the thermal insulation properties of the bryophyte layer, which will depend on the species and its thickness (Gornall, Jónsdóttir, Woodin, & Van Der Wal, 2007; Walker et al., 2003). ALT may also have indirect effects on nonvascular communities by benefiting vascular plants, which can reach nutrients that are available at greater soil depth with their root system and, thus, outcompete nonvascular species, which are limited to the surface, for mineral resources (Keuper et al., 2012; Wang et al., 2016).

### 4.5 Vegetation cover‐species diversity relations

Vegetation cover and species diversity were related according to our results, although the relationship was different depending on site and PFT. On the lakebed, vascular cover increased with increasing species diversity. In communities where the two dominant species on the lakebed (*B. nana* and *E. angustifolium*) form sparser canopies, higher number of species can coexist because of reduced shading. The nondominant species occupy different layers within the canopy, overlapping in many cases. These layers might explain the positive correlation between cover and diversity, as the point quadrat method we used allows us to take the vertical canopy structure into account. However, on the ridge and contrary to our hypothesis, vascular cover was higher in less diverse communities. This negative correlation might be explained by two main factors. On one hand, communities with the lowest species diversity are found in sedge wet hollows, which are dominated generally by *E. angustifolium*. This species grows faster and produces more biomass than the other species, resulting in higher cover and litter deposition, which limit the number of coexisting species due to resource and light competition. Additionally, the depressions where those communities grow accumulate greater amounts of snow compared to other habitats, allowing higher individuals than in more diverse communities. On the other hand, we might have underestimated the vascular cover of diverse communities on the ridge (as well as the nonvascular cover) due to the small size of the individuals comprising these communities in relation to the rather coarse grid cells of our sampling scheme.

Aboveground biomass has been widely used as a proxy of productivity (Chiarucci, Wilson, Anderson, & De Dominicis, 1999; Fraser et al., 2015). In tundra, aboveground biomass and production are highly correlated (Webber, 1978). Furthermore, vegetation cover and aboveground biomass were correlated in different ecosystems (Grytnes, 2000; Zhang, Cui, Shen, & Liu, 2016). These correlations and our results for the lakebed suggest that primary productivity and species diversity are positively related, supporting previous findings, although not in the case of the ridge (Hooper et al., 2005; Loreau et al., 2001; Tilman, Wedin, & Knops, 1996). This relationship inconsistency between sites may be attributed to complex mechanisms controlling species diversity and productivity (Grace et al., 2016). Further efforts to improve the vegetation cover estimation and explore the actual relations among cover, biomass, and productivity in the study area will provide a better understanding of the species diversity role in ecosystem functioning in Siberian tundra.

### 4.6 Climate change and tundra vegetation

Air temperature and precipitation are projected to increase in the Arctic by around 3°C and 20%, respectively (emission scenario RCP4.5, IPCC 2013). How these changes will propagate into edaphic factors, such as soil temperature, moisture, or active layer thickness, remains uncertain in terms of direction and spatial variability (Walwoord & Kurylyk, 2016). Our study highlights that tundra vegetation is closely related to a combination of edaphic factors going beyond soil acidity and moisture only. It is therefore important to increase our understanding of how atmospheric changes propagate into edaphic factors for more reliable predictions of vegetation shifts.

In addition to edaphic factors, several complex interactions and buffering mechanisms (e.g., competition and facilitation) will determine species diversity and community composition (Chapin & Shaver, 1996). For example, under a scenario of increased soil temperature and moisture, mineralization rates of soil organic matter are expected to rise, resulting in a higher nutrient availability (Schmidt, Jonasson, & Michelsen, 1999). In addition, the release of organic acids during the decomposition process will increase soil acidity (Satchel, 1974). According to our results, nonvascular diversity may decrease due to these expected changes in edaphic factors, while vascular diversity may remain constant. Shifts in community composition may also be caused by direct (e.g., changes in soil conditions) or indirect effects. For instance, soil warming and higher nutrient availability may increase the height and abundance of graminoids and shrubs, which will outcompete nonvascular species (Hudson, Henry, & Cornwell, 2011). These vegetation shifts will result in feedbacks to energy fluxes, permafrost thawing, and soil conditions, which may stabilize or accelerate those changes. In case of permafrost thawing, increasing soil temperature may promote a rise in active layer thickness (Ritcher‐Menge & Overland, 2010), but vegetation shifts, such as an expansion of shrub cover, may protect permafrost from thawing through soil shading (Blok et al., 2010; Nauta et al., 2014). On the other hand, an increasing decomposition rate may lead to a decrease of standing litter in wet sedge‐dominated areas, resulting in less soil shading (Juszak, Eugster, Heijmans, & Schaepman‐Strub, 2016).

In summary, because of uncertainties in responses of edaphic factors to projected climatic conditions and complex feedbacks involved in vegetation shifts, species diversity and community composition predictions based only on changes of edaphic factors may be highly inaccurate. However, identifying the vegetation responses to edaphic factors is a first step for a better understanding of vegetation–soil feedbacks under climate change. The distinct relationships found for nonvascular and vascular species suggest the importance of considering multiple PFT for predicting vegetation shifts, primary productivity, and in turn, ecosystem functioning in a changing climate.

## Conflict of Interest

None declared.
