# Supplementary material for: Interactive effects between plant functional types and soil factors on tundra species diversity and community composition
Source: Ecol Evol. 2016 Oct 17;6(22):8126–37. doi: 10.1002/ece3.2548 (PMC5108264; doi:10.1002/ece3.2548)
Supplement: Supplementary file 1 [file ECE3-6-8126-s001.docx]

**Supporting information**

Figure S1: Species richness (left) and Shannon index (right) on lakebed and ridge for nonvascular (open circles) and vascular (open triangles) plant functional types of the 40 selected plots. Close circles and triangles represent the mean diversity predicted by the model for both PFT. Error bars are the standard error of the predicted values.

Table S1a: Species name including the authority, abbreviation and species abundance on the ridge plots.

| **Species name** | **Abbreviation** | **P01** | **P02** | **P03** | **P04** | **P05** | **P06** | **P07** | **P08** | **P09** | **P10** | **P11** | **P12** | **P13** | **P14** | **P15** | **P31** | **P32** | **P33** | **P34** | **P35** |
| --- | --- | --- | --- | --- | --- | --- | --- | --- | --- | --- | --- | --- | --- | --- | --- | --- | --- | --- | --- | --- | --- |
| **Shrubs** |  |  |  |  |  |  |  |  |  |  |  |  |  |  |  |  |  |  |  |  |  |
| *Arctous alpina* (L.) Nied. | Arctalpi | 0 | 0 | 25 | 0 | 0 | 0 | 0 | 0 | 0 | 0 | 0 | 0 | 0 | 0 | 0 | 0 | 18 | 0 | 0 | 0 |
| *Betula nana* ssp. *exilis* (Sukazcev) Hultén | Betunana | 8 | 16 | 1 | 15 | 22 | 15 | 9 | 13 | 16 | 18 | 1 | 16 | 15 | 2 | 5 | 14 | 7 | 18 | 16 | 14 |
| *Cassiope tetragona* (L.) D. Don. | Casstetr | 0 | 0 | 0 | 0 | 0 | 0 | 0 | 0 | 0 | 0 | 0 | 2 | 0 | 0 | 20 | 0 | 0 | 0 | 0 | 17 |
| *Dryas octopetala* L. | Dryaocto | 0 | 3 | 12 | 0 | 0 | 0 | 0 | 0 | 0 | 0 | 3 | 1 | 0 | 0 | 3 | 0 | 3 | 0 | 0 | 0 |
| *Ledum palustre* ssp. *decumbens* (Aiton) Hultén | Ledupalu | 21 | 20 | 19 | 22 | 22 | 23 | 0 | 19 | 24 | 18 | 20 | 25 | 19 | 25 | 23 | 0 | 23 | 19 | 16 | 20 |
| *Salix fuscenses* Andersson | Salifusc | 0 | 0 | 0 | 0 | 0 | 0 | 0 | 0 | 0 | 0 | 0 | 0 | 0 | 0 | 0 | 0 | 0 | 0 | 0 | 0 |
| *Salix glauca* L. | Saliglau | 0 | 0 | 0 | 0 | 0 | 0 | 0 | 0 | 0 | 0 | 1 | 0 | 0 | 0 | 0 | 0 | 0 | 0 | 0 | 0 |
| *Salix pulchra* Cham. | Salipulc | 0 | 1 | 0 | 3 | 6 | 2 | 11 | 3 | 0 | 2 | 0 | 4 | 1 | 1 | 0 | 15 | 0 | 7 | 3 | 0 |
| *Vaccinium uliginosum* L. | Vacculig | 0 | 8 | 0 | 12 | 0 | 0 | 0 | 1 | 0 | 0 | 10 | 0 | 0 | 0 | 0 | 3 | 1 | 0 | 18 | 0 |
| *Vaccinium vitis-idaea* L. | Vaccviti | 24 | 25 | 25 | 25 | 25 | 25 | 0 | 23 | 25 | 25 | 25 | 25 | 24 | 25 | 25 | 0 | 25 | 22 | 0 | 25 |
| **Forbs** |  |  |  |  |  |  |  |  |  |  |  |  |  |  |  |  |  |  |  |  |  |
| *Petasites frigidus* (L.) Fr. | Petafrig | 0 | 0 | 0 | 0 | 0 | 0 | 0 | 0 | 1 | 0 | 0 | 0 | 0 | 0 | 0 | 0 | 0 | 0 | 0 | 0 |
| *Parrya nudicaulis* (L.) Boiss. | Parrnudi | 1 | 0 | 0 | 0 | 0 | 0 | 0 | 0 | 0 | 0 | 0 | 0 | 0 | 0 | 0 | 0 | 1 | 0 | 0 | 0 |
| *Pedicularis capitata* Adams | Pedicapi | 0 | 0 | 0 | 0 | 0 | 0 | 0 | 0 | 0 | 0 | 0 | 0 | 0 | 0 | 0 | 0 | 1 | 0 | 0 | 0 |
| *Pedicularis lapponica* L. | Pedilapp | 0 | 0 | 0 | 0 | 0 | 0 | 0 | 0 | 0 | 0 | 0 | 0 | 5 | 0 | 0 | 0 | 0 | 0 | 0 | 0 |
| *Pyrola grandiflora* Radius | Pyrogran | 0 | 3 | 0 | 0 | 0 | 0 | 0 | 0 | 0 | 0 | 0 | 0 | 0 | 0 | 0 | 0 | 0 | 0 | 0 | 0 |
| *Saxifraga nelsoniana* D. Don | Saxinels | 0 | 0 | 0 | 0 | 0 | 0 | 0 | 0 | 3 | 0 | 0 | 0 | 0 | 1 | 2 | 0 | 2 | 0 | 0 | 0 |
| *Valeriana capitata* Pall. ex Link | Valecapi | 3 | 2 | 0 | 0 | 1 | 0 | 0 | 0 | 0 | 0 | 1 | 1 | 0 | 0 | 2 | 0 | 5 | 0 | 0 | 0 |
| **Graminoids** |  |  |  |  |  |  |  |  |  |  |  |  |  |  |  |  |  |  |  |  |  |
| *Arctagrostis latifolia* (R.Br.) Griseb. | Arctlati | 12 | 2 | 0 | 2 | 11 | 6 | 0 | 0 | 0 | 0 | 0 | 0 | 0 | 0 | 0 | 0 | 1 | 3 | 4 | 3 |
| *Calamagrostis holmii* Lange | Calaholm | 0 | 9 | 16 | 15 | 16 | 20 | 0 | 9 | 11 | 6 | 1 | 10 | 9 | 7 | 16 | 0 | 5 | 0 | 6 | 5 |
| *Carex aquatilis* var. *minor* Boott | Careaqua | 0 | 0 | 0 | 0 | 0 | 0 | 0 | 0 | 0 | 6 | 0 | 0 | 0 | 0 | 0 | 0 | 0 | 0 | 0 | 4 |
| *Carex bigelowii* ssp. *arctisibirica* (Jurtzev) Á.& D.Löve | Carebige | 0 | 22 | 21 | 0 | 8 | 3 | 0 | 9 | 0 | 0 | 20 | 2 | 9 | 10 | 12 | 0 | 21 | 0 | 0 | 0 |
| *Eriophorum angustifolium* Honck. | Erioangu | 0 | 0 | 0 | 0 | 0 | 0 | 25 | 0 | 0 | 0 | 0 | 0 | 0 | 0 | 0 | 25 | 0 | 0 | 0 | 0 |
| *Eriophorum vaginatum* L. | Eriovagi | 9 | 0 | 0 | 12 | 2 | 1 | 0 | 23 | 22 | 19 | 3 | 10 | 10 | 13 | 5 | 0 | 0 | 25 | 17 | 20 |
| *Luzula nivalis* (Laest.) Spreng. | Luzuniva | 1 | 0 | 0 | 0 | 0 | 0 | 0 | 0 | 0 | 0 | 0 | 0 | 0 | 0 | 0 | 0 | 0 | 0 | 0 | 0 |
| *Luzula wahlenbergii* Rupr. | Luzuwahl | 0 | 0 | 0 | 0 | 0 | 0 | 0 | 0 | 0 | 0 | 0 | 0 | 0 | 0 | 0 | 0 | 0 | 0 | 0 | 0 |
| **Bryophytes** |  |  |  |  |  |  |  |  |  |  |  |  |  |  |  |  |  |  |  |  |  |
| *Aulacomnium palustre* (Hedw.) Schwägr. | Aulapalu | 0 | 0 | 24 | 12 | 8 | 24 | 11 | 11 | 24 | 12 | 2 | 16 | 3 | 7 | 4 | 0 | 10 | 10 | 6 | 12 |
| *Aulacomnium turgidum* (Wahlenb.) Schwägr. | Aulaturg | 0 | 22 | 8 | 0 | 2 | 0 | 0 | 0 | 0 | 8 | 0 | 16 | 0 | 23 | 3 | 0 | 2 | 0 | 0 | 0 |
| *Blepharostoma trichophyllum* (L.) Dumort. | Bleptric | 0 | 0 | 0 | 0 | 0 | 0 | 0 | 0 | 0 | 0 | 0 | 0 | 0 | 0 | 0 | 0 | 0 | 0 | 0 | 0 |
| *Brachythecium* sp. | Bracsp | 0 | 0 | 0 | 0 | 0 | 0 | 0 | 0 | 0 | 0 | 0 | 0 | 0 | 0 | 0 | 0 | 0 | 0 | 1 | 0 |
| *Dicranum elongatum* Schleich. ex Schwägr. | Dicrelon | 20 | 4 | 6 | 17 | 15 | 0 | 0 | 23 | 12 | 0 | 16 | 15 | 20 | 25 | 20 | 0 | 0 | 0 | 0 | 0 |
| *Dicranum spadiceum* J.E. Zetterst | Dicrspad | 20 | 4 | 0 | 0 | 15 | 23 | 0 | 0 | 12 | 25 | 16 | 15 | 20 | 0 | 0 | 0 | 0 | 0 | 0 | 0 |
| *Dicranum* sp. | Dicrsp | 0 | 0 | 0 | 0 | 0 | 0 | 0 | 0 | 0 | 0 | 0 | 0 | 0 | 0 | 0 | 0 | 7 | 0 | 0 | 12 |
| *Hylocomium splendens* (Hedw.) Schimp. | Hylosple | 4 | 25 | 13 | 23 | 10 | 18 | 0 | 0 | 15 | 5 | 23 | 22 | 9 | 0 | 3 | 0 | 0 | 10 | 0 | 8 |
| *Polytrichastrum alpinum* (Hedw.) G.L. Sm. | Polyalpi | 0 | 8 | 0 | 0 | 0 | 0 | 0 | 0 | 0 | 0 | 0 | 0 | 0 | 0 | 0 | 0 | 0 | 0 | 0 | 0 |
| *Polytrichum hyperboreum* R. Br. | Polyhype | 1 | 0 | 0 | 0 | 0 | 0 | 0 | 0 | 0 | 0 | 0 | 0 | 0 | 0 | 9 | 0 | 0 | 0 | 0 | 0 |
| *Polytrichum jensenii* I. Hagen | Polyjens | 0 | 0 | 0 | 0 | 0 | 0 | 0 | 7 | 0 | 3 | 0 | 0 | 0 | 0 | 0 | 0 | 0 | 0 | 0 | 0 |
| *Polytrichum juniperinum* Hedw. | Polyjuni | 0 | 0 | 17 | 7 | 3 | 0 | 0 | 0 | 3 | 0 | 3 | 0 | 2 | 2 | 0 | 0 | 16 | 0 | 0 | 0 |
| *Polytrichum piliferum* Hedw. | Polypili | 0 | 0 | 0 | 0 | 0 | 0 | 0 | 11 | 0 | 0 | 0 | 0 | 0 | 0 | 0 | 0 | 0 | 0 | 0 | 0 |
| *Ptilidium ciliare* (L.) Hampe | Ptilcili | 0 | 2 | 5 | 0 | 21 | 0 | 0 | 0 | 0 | 7 | 0 | 10 | 5 | 0 | 0 | 0 | 0 | 0 | 14 | 2 |
| *Sanionia uncinata* (Hedw.) Loeske | Saniunci | 0 | 0 | 0 | 0 | 0 | 0 | 0 | 0 | 0 | 0 | 1 | 0 | 0 | 0 | 0 | 0 | 0 | 0 | 0 | 0 |
| *Sphagnum balticum* (Russow) C.E.O. Jensen | Sphabalt | 0 | 0 | 0 | 0 | 0 | 0 | 0 | 9 | 0 | 0 | 0 | 0 | 0 | 0 | 0 | 0 | 0 | 0 | 0 | 0 |
| *Sphagnum compactum* Lam. & DC. | Sphacomp | 0 | 0 | 0 | 0 | 0 | 0 | 0 | 0 | 0 | 0 | 0 | 0 | 0 | 0 | 0 | 0 | 0 | 5 | 15 | 0 |
| *Sphagnum lenense* Pohle | Sphalene | 0 | 0 | 0 | 0 | 0 | 0 | 0 | 0 | 0 | 1 | 0 | 0 | 0 | 0 | 0 | 0 | 0 | 0 | 0 | 0 |
| *Sphagnum obtusum* Warnst. | Sphaobtu | 0 | 0 | 0 | 0 | 0 | 0 | 19 | 0 | 0 | 0 | 0 | 0 | 0 | 0 | 0 | 0 | 0 | 0 | 0 | 0 |
| *Sphagnum squarrosum* Crome | Sphasqua | 0 | 0 | 0 | 0 | 0 | 0 | 0 | 0 | 0 | 0 | 0 | 0 | 0 | 0 | 0 | 4 | 0 | 0 | 0 | 0 |
| *Sphagnum teres* (Schimp.) Ångström | Sphatere | 0 | 0 | 0 | 0 | 0 | 0 | 0 | 23 | 0 | 0 | 0 | 0 | 0 | 0 | 0 | 0 | 0 | 0 | 0 | 0 |
| *Sphenolobus minutus* (Schreb.) Berggr. | Spheminu | 20 | 4 | 6 | 20 | 15 | 23 | 0 | 14 | 12 | 25 | 16 | 15 | 20 | 25 | 20 | 0 | 7 | 0 | 0 | 12 |
| *Tomentypnum nitens* (Hedw.) Loeske | Tomenite | 21 | 0 | 18 | 1 | 24 | 18 | 3 | 0 | 2 | 12 | 22 | 9 | 10 | 0 | 23 | 0 | 7 | 12 | 18 | 16 |
| *Tritomaria exsectiformis* (Breidl.) Schiffner ex Loeske | Tritexse | 0 | 0 | 0 | 0 | 0 | 0 | 0 | 0 | 0 | 0 | 0 | 0 | 0 | 0 | 0 | 0 | 0 | 0 | 0 | 0 |
|  |  |  |  |  |  |  |  |  |  |  |  |  |  |  |  |  |  |  |  |  |  |

(continuation)

| **Species name** | **Abbreviation** | **P01** | **P02** | **P03** | **P04** | **P05** | **P06** | **P07** | **P08** | **P09** | **P10** | **P11** | **P12** | **P13** | **P14** | **P15** | **P31** | **P32** | **P33** | **P34** | **P35** |
| --- | --- | --- | --- | --- | --- | --- | --- | --- | --- | --- | --- | --- | --- | --- | --- | --- | --- | --- | --- | --- | --- |
| **Lichens** |  |  |  |  |  |  |  |  |  |  |  |  |  |  |  |  |  |  |  |  |  |
| *Alectoria nigricans* (Ach.) Nyl | Alecnigr | 0 | 0 | 0 | 0 | 0 | 0 | 0 | 0 | 0 | 0 | 0 | 0 | 0 | 0 | 0 | 0 | 5 | 0 | 0 | 0 |
| *Cetraria ericetorum* Opiz | Cetreric | 0 | 0 | 0 | 16 | 0 | 0 | 0 | 0 | 0 | 0 | 0 | 0 | 0 | 0 | 0 | 0 | 0 | 0 | 0 | 0 |
| *Cetraria islandica* (L.) Ach. | Cetrisla | 20 | 7 | 22 | 8 | 0 | 10 | 0 | 0 | 5 | 0 | 22 | 8 | 10 | 24 | 19 | 0 | 17 | 0 | 0 | 7 |
| *Cladonia amaurocraea* (Florke) Schaerer | Cladamau | 0 | 0 | 0 | 2 | 0 | 0 | 0 | 0 | 0 | 0 | 0 | 0 | 0 | 0 | 0 | 0 | 0 | 0 | 0 | 0 |
| *Cladonia borealis* S. Stenroos | Cladbore | 0 | 0 | 0 | 0 | 0 | 0 | 0 | 0 | 0 | 0 | 0 | 2 | 0 | 0 | 0 | 0 | 0 | 0 | 0 | 0 |
| *Cladonia cenotea* (Ach.) Schaerer | Cladceno | 0 | 0 | 0 | 0 | 0 | 0 | 0 | 0 | 0 | 0 | 0 | 0 | 0 | 0 | 0 | 0 | 0 | 0 | 0 | 0 |
| *Cladonia chlorophaea* (Florke ex Sommerf.) Sprengel | Cladchlo | 0 | 0 | 0 | 0 | 0 | 0 | 0 | 0 | 0 | 0 | 4 | 0 | 0 | 0 | 0 | 0 | 0 | 0 | 0 | 0 |
| *Cladonia coccifera* (L.) Willd. | Cladcocc | 0 | 0 | 2 | 5 | 0 | 0 | 0 | 0 | 0 | 0 | 5 | 0 | 0 | 0 | 0 | 0 | 5 | 0 | 0 | 0 |
| *Cladonia cornuta* (L.) Hoffm. | Cladcorn | 0 | 0 | 0 | 0 | 0 | 0 | 0 | 0 | 0 | 0 | 0 | 0 | 0 | 0 | 0 | 0 | 1 | 0 | 0 | 0 |
| *Cladonia cyanipes* (Sommerf.) Nyl. | Cladcyan | 0 | 0 | 0 | 0 | 0 | 0 | 0 | 0 | 0 | 0 | 0 | 0 | 0 | 0 | 0 | 0 | 2 | 0 | 0 | 5 |
| *Cladonia deformis* (L.) Hoffm. | Claddefo | 0 | 0 | 0 | 0 | 0 | 0 | 0 | 0 | 0 | 0 | 0 | 0 | 0 | 0 | 0 | 0 | 0 | 0 | 0 | 0 |
| *Cladonia ecmocyna* Leighton | Cladecmo | 0 | 0 | 0 | 0 | 0 | 0 | 0 | 0 | 0 | 0 | 0 | 0 | 1 | 0 | 0 | 0 | 0 | 0 | 0 | 0 |
| *Cladonia gracilis* (L.) Willd. | Cladgrac | 0 | 4 | 4 | 0 | 0 | 0 | 0 | 0 | 0 | 0 | 0 | 0 | 0 | 0 | 3 | 0 | 12 | 0 | 0 | 6 |
| *Cladonia macroceras* (Delise) Hav. | Cladmacr | 0 | 0 | 0 | 1 | 0 | 0 | 0 | 0 | 0 | 0 | 0 | 0 | 1 | 0 | 0 | 0 | 0 | 0 | 0 | 0 |
| *Cladonia pleurota* (Florke) Schaerer | Cladpleu | 0 | 0 | 0 | 0 | 0 | 0 | 0 | 0 | 0 | 0 | 0 | 0 | 0 | 0 | 0 | 0 | 0 | 0 | 0 | 0 |
| *Cladonia rangiferina* (L.) F. H. Wigg. | Cladrang | 13 | 0 | 0 | 0 | 0 | 0 | 0 | 0 | 0 | 0 | 0 | 0 | 0 | 0 | 0 | 0 | 0 | 0 | 0 | 0 |
| *Cladonia sulphurina* (Michaux) Fr. | Cladsulp | 0 | 0 | 0 | 0 | 0 | 0 | 0 | 0 | 0 | 0 | 1 | 0 | 0 | 0 | 0 | 0 | 0 | 0 | 0 | 0 |
| *Dactylina arctica* (Richardson) Nyl. | Dactarct | 18 | 0 | 15 | 2 | 0 | 0 | 0 | 13 | 0 | 0 | 4 | 0 | 0 | 0 | 1 | 0 | 20 | 0 | 0 | 7 |
| *Flavocetraria cucullata* (Bellardi) Karnefelt & Thell | Flavcucu | 24 | 16 | 23 | 0 | 0 | 8 | 0 | 0 | 17 | 2 | 25 | 21 | 17 | 25 | 25 | 0 | 23 | 25 | 0 | 3 |
| *Nephroma expallidum (*Nyl.) Nyl. | Nephexpa | 0 | 0 | 0 | 0 | 0 | 0 | 0 | 0 | 0 | 0 | 0 | 3 | 0 | 0 | 0 | 0 | 0 | 0 | 0 | 0 |
| *Ochrolechia inaequatula* (Nyl.) Zahlbr. | Ochrinae | 0 | 3 | 0 | 0 | 3 | 0 | 0 | 0 | 0 | 0 | 0 | 0 | 3 | 0 | 4 | 0 | 5 | 0 | 0 | 7 |
| *Peltigera aphtosa* (L.) Willd. | Peltapht | 9 | 0 | 13 | 0 | 16 | 0 | 0 | 0 | 0 | 0 | 16 | 7 | 7 | 2 | 17 | 0 | 9 | 10 | 4 | 16 |
| *Peltigera frippii* Holt.-Hartw. | Peltfrip | 0 | 0 | 2 | 4 | 1 | 0 | 0 | 0 | 0 | 0 | 7 | 0 | 0 | 0 | 0 | 0 | 0 | 0 | 0 | 0 |
| *Peltigera leucophlebia* (Nyl.) Gyelnik | Peltleuc | 0 | 19 | 0 | 15 | 0 | 0 | 0 | 0 | 0 | 12 | 0 | 0 | 0 | 0 | 2 | 0 | 0 | 0 | 8 | 0 |
| *Peltigera occidentalis* (Å.E. Dahl) Kristinsson | Peltocci | 0 | 0 | 0 | 0 | 0 | 0 | 0 | 1 | 0 | 0 | 0 | 0 | 0 | 0 | 0 | 0 | 0 | 0 | 0 | 0 |
| *Peltigera scabrosa* Th. Fr. | Peltscab | 8 | 0 | 0 | 0 | 5 | 8 | 0 | 2 | 9 | 0 | 0 | 0 | 3 | 0 | 0 | 0 | 0 | 0 | 0 | 0 |
| *Protopannaria pezizoides* (Weber) P.M.Joerg.&S.Ekman | Protpezi | 0 | 0 | 0 | 0 | 0 | 0 | 0 | 0 | 0 | 0 | 0 | 0 | 0 | 0 | 0 | 0 | 0 | 0 | 0 | 0 |
| *Stereocaulon alpinum* Laurer ex Funck | Steralpi | 0 | 0 | 1 | 0 | 0 | 0 | 0 | 0 | 0 | 0 | 0 | 0 | 0 | 0 | 0 | 0 | 0 | 0 | 0 | 0 |
| *Thamnolia vermicularis* (Sw.) Ach. ex Schaerer | Thamverm | 0 | 0 | 3 | 0 | 0 | 0 | 0 | 0 | 0 | 0 | 0 | 0 | 0 | 0 | 0 | 0 | 2 | 0 | 0 | 0 |
|  |  |  |  |  |  |  |  |  |  |  |  |  |  |  |  |  |  |  |  |  |  |

Table S1b: Species name including the authority, abbreviation and species abundance on the lakebed plots.

| **Species name** | **Abbreviation** | **P16** | **P17** | **P18** | **P19** | **P20** | **P21** | **P22** | **P23** | **P24** | **P25** | **P26** | **P27** | **P28** | **P29** | **P30** | **P36** | **P37** | **P38** | **P39** | **P40** |
| --- | --- | --- | --- | --- | --- | --- | --- | --- | --- | --- | --- | --- | --- | --- | --- | --- | --- | --- | --- | --- | --- |
| **Shrubs** |  |  |  |  |  |  |  |  |  |  |  |  |  |  |  |  |  |  |  |  |  |
| *Arctous alpina* (L.) Nied. | Arctalpi | 0 | 0 | 0 | 0 | 0 | 0 | 0 | 0 | 0 | 0 | 0 | 0 | 0 | 0 | 0 | 0 | 0 | 0 | 0 | 0 |
| *Betula nana* ssp. *exilis* (Sukazcev) Hultén | Betunana | 0 | 22 | 3 | 24 | 20 | 22 | 22 | 0 | 11 | 12 | 21 | 19 | 0 | 12 | 23 | 12 | 13 | 21 | 22 | 25 |
| *Cassiope tetragona* (L.) D. Don. | Casstetr | 0 | 0 | 0 | 0 | 0 | 0 | 0 | 0 | 0 | 0 | 0 | 0 | 0 | 0 | 0 | 0 | 0 | 0 | 0 | 0 |
| *Dryas octopetala* L. | Dryaocto | 0 | 0 | 0 | 0 | 0 | 0 | 0 | 0 | 0 | 0 | 0 | 0 | 0 | 0 | 0 | 0 | 0 | 0 | 0 | 0 |
| *Ledum palustre* ssp. *decumbens* (Aiton) Hultén | Ledupalu | 0 | 0 | 0 | 0 | 0 | 0 | 0 | 0 | 0 | 0 | 0 | 4 | 0 | 0 | 0 | 0 | 0 | 12 | 0 | 0 |
| *Salix fuscenses* Andersson | Salifusc | 0 | 0 | 0 | 0 | 0 | 0 | 0 | 0 | 0 | 0 | 0 | 0 | 5 | 0 | 0 | 0 | 13 | 0 | 0 | 0 |
| *Salix glauca* L. | Saliglau | 0 | 0 | 0 | 0 | 0 | 0 | 0 | 0 | 0 | 0 | 0 | 0 | 0 | 0 | 0 | 0 | 0 | 0 | 0 | 0 |
| *Salix pulchra* Cham. | Salipulc | 0 | 0 | 0 | 7 | 0 | 0 | 0 | 0 | 2 | 3 | 0 | 0 | 14 | 11 | 1 | 1 | 3 | 0 | 12 | 6 |
| *Vaccinium uliginosum* L. | Vacculig | 0 | 0 | 0 | 0 | 0 | 0 | 0 | 0 | 0 | 0 | 0 | 0 | 0 | 0 | 0 | 0 | 0 | 0 | 0 | 0 |
| *Vaccinium vitis-idaea* L. | Vaccviti | 0 | 0 | 0 | 0 | 0 | 0 | 0 | 0 | 25 | 0 | 0 | 25 | 0 | 8 | 0 | 25 | 0 | 25 | 25 | 18 |
| **Forbs** |  |  |  |  |  |  |  |  |  |  |  |  |  |  |  |  |  |  |  |  |  |
| *Petasites frigidus* (L.) Fr. | Petafrig | 0 | 0 | 0 | 0 | 0 | 0 | 0 | 0 | 0 | 0 | 0 | 0 | 0 | 0 | 0 | 0 | 0 | 0 | 0 | 0 |
| *Parrya nudicaulis* (L.) Boiss. | Parrnudi | 0 | 0 | 0 | 0 | 0 | 0 | 0 | 0 | 0 | 0 | 0 | 0 | 0 | 0 | 0 | 0 | 0 | 0 | 0 | 0 |
| *Pedicularis capitata* Adams | Pedicapi | 0 | 0 | 0 | 0 | 0 | 0 | 0 | 0 | 0 | 0 | 0 | 0 | 0 | 0 | 0 | 0 | 0 | 0 | 0 | 0 |
| *Pedicularis lapponica* L. | Pedilapp | 0 | 0 | 0 | 6 | 0 | 0 | 0 | 0 | 0 | 0 | 0 | 0 | 0 | 0 | 0 | 0 | 0 | 0 | 1 | 12 |
| *Pyrola grandiflora* Radius | Pyrogran | 0 | 0 | 0 | 0 | 0 | 0 | 0 | 0 | 0 | 0 | 0 | 0 | 0 | 0 | 0 | 0 | 0 | 0 | 0 | 0 |
| *Saxifraga nelsoniana* D. Don | Saxinels | 0 | 0 | 0 | 0 | 0 | 0 | 0 | 0 | 0 | 0 | 0 | 0 | 8 | 0 | 0 | 0 | 0 | 0 | 0 | 0 |
| *Valeriana capitata* Pall. ex Link | Valecapi | 0 | 0 | 0 | 0 | 0 | 0 | 0 | 0 | 0 | 0 | 0 | 0 | 0 | 0 | 0 | 0 | 0 | 0 | 0 | 0 |
| **Graminoids** |  |  |  |  |  |  |  |  |  |  |  |  |  |  |  |  |  |  |  |  |  |
| *Arctagrostis latifolia* (R.Br.) Griseb. | Arctlati | 0 | 0 | 0 | 0 | 0 | 0 | 0 | 0 | 0 | 0 | 0 | 9 | 0 | 0 | 0 | 6 | 0 | 0 | 0 | 0 |
| *Calamagrostis holmii* Lange | Calaholm | 0 | 24 | 0 | 19 | 21 | 19 | 20 | 0 | 25 | 10 | 6 | 17 | 2 | 5 | 0 | 8 | 5 | 16 | 23 | 3 |
| *Carex aquatilis* var. *minor* Boott | Careaqua | 0 | 0 | 0 | 0 | 0 | 0 | 0 | 0 | 0 | 0 | 0 | 0 | 8 | 18 | 0 | 0 | 0 | 0 | 12 | 0 |
| *Carex bigelowii* ssp. *arctisibirica* (Jurtzev) Á.& D.Löve | Carebige | 0 | 0 | 0 | 0 | 0 | 0 | 0 | 0 | 0 | 0 | 0 | 0 | 0 | 0 | 0 | 0 | 0 | 0 | 0 | 0 |
| *Eriophorum angustifolium* Honck. | Erioangu | 25 | 0 | 25 | 0 | 0 | 1 | 0 | 25 | 0 | 0 | 0 | 0 | 25 | 8 | 18 | 0 | 23 | 0 | 0 | 19 |
| *Eriophorum vaginatum* L. | Eriovagi | 0 | 0 | 0 | 0 | 0 | 3 | 0 | 0 | 0 | 0 | 0 | 2 | 0 | 1 | 1 | 0 | 0 | 0 | 0 | 0 |
| *Luzula nivalis* (Laest.) Spreng. | Luzuniva | 0 | 0 | 0 | 0 | 0 | 0 | 0 | 0 | 0 | 0 | 0 | 0 | 0 | 0 | 0 | 0 | 0 | 0 | 0 | 0 |
| *Luzula wahlenbergii* Rupr. | Luzuwahl | 0 | 0 | 0 | 0 | 0 | 0 | 0 | 0 | 0 | 0 | 0 | 0 | 0 | 2 | 0 | 0 | 0 | 0 | 0 | 0 |
| **Bryophytes** |  |  |  |  |  |  |  |  |  |  |  |  |  |  |  |  |  |  |  |  |  |
| *Aulacomnium palustre* (Hedw.) Schwägr. | Aulapalu | 0 | 5 | 0 | 0 | 0 | 0 | 0 | 0 | 3 | 1 | 0 | 0 | 21 | 3 | 0 | 0 | 0 | 0 | 0 | 0 |
| *Aulacomnium turgidum* (Wahlenb.) Schwägr. | Aulaturg | 0 | 19 | 0 | 23 | 16 | 0 | 4 | 0 | 6 | 17 | 11 | 2 | 0 | 0 | 6 | 22 | 20 | 5 | 8 | 9 |
| *Blepharostoma trichophyllum* (L.) Dumort. | Bleptric | 0 | 0 | 0 | 11 | 0 | 0 | 0 | 0 | 0 | 0 | 0 | 0 | 0 | 0 | 0 | 0 | 0 | 0 | 0 | 0 |
| *Brachythecium* sp. | Bracsp | 0 | 0 | 0 | 0 | 0 | 0 | 0 | 0 | 0 | 0 | 0 | 0 | 0 | 0 | 0 | 0 | 0 | 0 | 0 | 0 |
| *Dicranum elongatum* Schleich. ex Schwägr. | Dicrelon | 0 | 19 | 0 | 0 | 0 | 0 | 19 | 0 | 0 | 0 | 0 | 23 | 0 | 0 | 0 | 0 | 0 | 0 | 0 | 0 |
| *Dicranum spadiceum* J.E. Zetterst | Dicrspad | 0 | 19 | 0 | 16 | 21 | 25 | 0 | 0 | 20 | 17 | 23 | 23 | 0 | 1 | 21 | 0 | 0 | 0 | 0 | 0 |
| *Dicranum* sp. | Dicrsp | 0 | 0 | 0 | 0 | 0 | 0 | 0 | 0 | 0 | 0 | 0 | 0 | 0 | 0 | 0 | 18 | 22 | 11 | 16 | 25 |
| *Hylocomium splendens* (Hedw.) Schimp. | Hylosple | 0 | 0 | 0 | 0 | 0 | 0 | 0 | 0 | 0 | 0 | 0 | 0 | 0 | 0 | 0 | 0 | 0 | 0 | 0 | 0 |
| *Polytrichastrum alpinum* (Hedw.) G.L. Sm. | Polyalpi | 0 | 0 | 0 | 21 | 0 | 0 | 0 | 0 | 13 | 0 | 0 | 0 | 0 | 4 | 0 | 0 | 0 | 0 | 0 | 0 |
| *Polytrichum hyperboreum* R. Br. | Polyhype | 0 | 25 | 0 | 21 | 0 | 0 | 0 | 0 | 22 | 0 | 0 | 0 | 0 | 0 | 0 | 0 | 0 | 0 | 0 | 0 |
| *Polytrichum jensenii* I. Hagen | Polyjens | 0 | 0 | 0 | 0 | 0 | 0 | 0 | 0 | 0 | 0 | 0 | 0 | 10 | 0 | 0 | 0 | 0 | 0 | 0 | 0 |
| *Polytrichum juniperinum* Hedw. | Polyjuni | 0 | 25 | 0 | 0 | 12 | 23 | 25 | 0 | 15 | 16 | 16 | 10 | 0 | 0 | 23 | 0 | 0 | 0 | 0 | 0 |
| *Polytrichum piliferum* Hedw. | Polypili | 0 | 0 | 0 | 0 | 0 | 0 | 0 | 0 | 0 | 0 | 0 | 0 | 0 | 0 | 0 | 0 | 0 | 0 | 0 | 0 |
| *Ptilidium ciliare* (L.) Hampe | Ptilcili | 0 | 0 | 0 | 3 | 0 | 3 | 2 | 0 | 0 | 3 | 3 | 0 | 0 | 0 | 0 | 0 | 0 | 7 | 0 | 14 |
| *Sanionia uncinata* (Hedw.) Loeske | Saniunci | 0 | 0 | 0 | 0 | 0 | 0 | 0 | 0 | 0 | 0 | 0 | 0 | 0 | 0 | 0 | 0 | 0 | 0 | 0 | 0 |
| *Sphagnum balticum* (Russow) C.E.O. Jensen | Sphabalt | 0 | 0 | 0 | 0 | 0 | 0 | 0 | 0 | 0 | 0 | 0 | 0 | 0 | 0 | 0 | 0 | 0 | 0 | 0 | 0 |
| *Sphagnum compactum* Lam. & DC. | Sphacomp | 0 | 0 | 0 | 5 | 0 | 0 | 0 | 0 | 0 | 0 | 0 | 0 | 0 | 0 | 0 | 0 | 0 | 0 | 0 | 0 |
| *Sphagnum lenense* Pohle | Sphalene | 0 | 0 | 0 | 0 | 0 | 0 | 0 | 0 | 0 | 0 | 0 | 0 | 0 | 0 | 0 | 0 | 0 | 0 | 0 | 0 |
| *Sphagnum obtusum* Warnst. | Sphaobtu | 0 | 0 | 25 | 0 | 0 | 0 | 0 | 25 | 0 | 0 | 0 | 0 | 10 | 25 | 0 | 0 | 15 | 0 | 23 | 18 |
| *Sphagnum squarrosum* Crome | Sphasqua | 0 | 0 | 0 | 0 | 0 | 0 | 0 | 0 | 0 | 0 | 0 | 0 | 0 | 0 | 0 | 0 | 0 | 0 | 0 | 0 |
| *Sphagnum teres* (Schimp.) Ångström | Sphatere | 0 | 0 | 0 | 0 | 0 | 2 | 0 | 0 | 0 | 0 | 0 | 0 | 0 | 0 | 0 | 0 | 0 | 0 | 0 | 0 |
| *Sphenolobus minutus* (Schreb.) Berggr. | Spheminu | 0 | 19 | 0 | 16 | 24 | 25 | 20 | 0 | 18 | 17 | 23 | 23 | 0 | 1 | 21 | 18 | 17 | 9 | 23 | 25 |
| *Tomentypnum nitens* (Hedw.) Loeske | Tomenite | 0 | 0 | 0 | 0 | 0 | 0 | 0 | 0 | 0 | 0 | 0 | 0 | 0 | 0 | 0 | 20 | 0 | 22 | 25 | 5 |
| *Tritomaria exsectiformis* (Breidl.) Schiffner ex Loeske | Tritexse | 0 | 0 | 0 | 0 | 0 | 0 | 0 | 0 | 0 | 0 | 0 | 0 | 0 | 2 | 0 | 0 | 0 | 0 | 0 | 0 |
|  |  |  |  |  |  |  |  |  |  |  |  |  |  |  |  |  |  |  |  |  |  |

(continuation)

| **Species name** | **Abbreviation** | **P16** | **P17** | **P18** | **P19** | **P20** | **P21** | **P22** | **P23** | **P24** | **P25** | **P26** | **P27** | **P28** | **P29** | **P30** | **P36** | **P37** | **P38** | **P39** | **P40** |
| --- | --- | --- | --- | --- | --- | --- | --- | --- | --- | --- | --- | --- | --- | --- | --- | --- | --- | --- | --- | --- | --- |
| **Lichens** |  |  |  |  |  |  |  |  |  |  |  |  |  |  |  |  |  |  |  |  |  |
| *Alectoria nigricans* (Ach.) Nyl | Alecnigr | 0 | 0 | 0 | 0 | 0 | 0 | 0 | 0 | 0 | 0 | 0 | 0 | 0 | 0 | 0 | 0 | 0 | 0 | 0 | 0 |
| *Cetraria ericetorum* Opiz | Cetreric | 0 | 0 | 0 | 4 | 0 | 0 | 0 | 0 | 0 | 0 | 0 | 0 | 0 | 0 | 0 | 0 | 0 | 0 | 0 | 0 |
| *Cetraria islandica* (L.) Ach. | Cetrisla | 0 | 7 | 0 | 0 | 0 | 0 | 14 | 0 | 10 | 2 | 7 | 22 | 0 | 0 | 8 | 13 | 0 | 3 | 3 | 6 |
| *Cladonia amaurocraea* (Florke) Schaerer | Cladamau | 0 | 0 | 0 | 4 | 0 | 0 | 2 | 0 | 0 | 0 | 0 | 0 | 0 | 0 | 0 | 0 | 0 | 0 | 0 | 0 |
| *Cladonia borealis* S. Stenroos | Cladbore | 0 | 1 | 0 | 0 | 0 | 0 | 0 | 0 | 0 | 0 | 0 | 0 | 0 | 0 | 0 | 0 | 0 | 0 | 0 | 0 |
| *Cladonia cenotea* (Ach.) Schaerer | Cladceno | 0 | 0 | 0 | 0 | 1 | 0 | 0 | 0 | 0 | 0 | 0 | 0 | 0 | 0 | 0 | 0 | 0 | 0 | 0 | 0 |
| *Cladonia chlorophaea* (Florke ex Sommerf.) Sprengel | Cladchlo | 0 | 0 | 0 | 0 | 2 | 0 | 4 | 0 | 0 | 0 | 0 | 0 | 0 | 0 | 0 | 0 | 0 | 0 | 0 | 0 |
| *Cladonia coccifera* (L.) Willd. | Cladcocc | 0 | 1 | 0 | 0 | 0 | 0 | 0 | 0 | 0 | 0 | 0 | 0 | 0 | 0 | 0 | 0 | 0 | 0 | 0 | 0 |
| *Cladonia cornuta* (L.) Hoffm. | Cladcorn | 0 | 0 | 0 | 0 | 0 | 0 | 0 | 0 | 0 | 0 | 0 | 0 | 0 | 0 | 0 | 0 | 0 | 0 | 0 | 0 |
| *Cladonia cyanipes* (Sommerf.) Nyl. | Cladcyan | 0 | 0 | 0 | 0 | 0 | 0 | 0 | 0 | 0 | 0 | 0 | 0 | 0 | 0 | 0 | 0 | 0 | 0 | 0 | 0 |
| *Cladonia deformis* (L.) Hoffm. | Claddefo | 0 | 0 | 0 | 0 | 0 | 0 | 0 | 0 | 0 | 0 | 2 | 0 | 0 | 0 | 0 | 0 | 0 | 0 | 0 | 0 |
| *Cladonia ecmocyna* Leighton | Cladecmo | 0 | 0 | 0 | 0 | 0 | 0 | 0 | 0 | 0 | 0 | 0 | 0 | 0 | 0 | 0 | 0 | 0 | 0 | 0 | 0 |
| *Cladonia gracilis* (L.) Willd. | Cladgrac | 0 | 1 | 0 | 0 | 0 | 0 | 0 | 0 | 0 | 2 | 4 | 1 | 0 | 0 | 0 | 0 | 0 | 4 | 0 | 0 |
| *Cladonia macroceras* (Delise) Hav. | Cladmacr | 0 | 0 | 0 | 0 | 0 | 0 | 0 | 0 | 0 | 2 | 0 | 0 | 0 | 0 | 0 | 0 | 0 | 0 | 0 | 0 |
| *Cladonia pleurota* (Florke) Schaerer | Cladpleu | 0 | 0 | 0 | 0 | 0 | 0 | 0 | 0 | 0 | 1 | 0 | 0 | 0 | 0 | 0 | 0 | 0 | 0 | 0 | 0 |
| *Cladonia rangiferina* (L.) F. H. Wigg. | Cladrang | 0 | 0 | 0 | 0 | 0 | 0 | 0 | 0 | 0 | 0 | 0 | 0 | 0 | 0 | 0 | 0 | 0 | 0 | 0 | 0 |
| *Cladonia sulphurina* (Michaux) Fr. | Cladsulp | 0 | 0 | 0 | 0 | 0 | 0 | 0 | 0 | 0 | 0 | 0 | 0 | 0 | 0 | 0 | 0 | 0 | 0 | 0 | 0 |
| *Dactylina arctica* (Richardson) Nyl. | Dactarct | 0 | 0 | 0 | 0 | 0 | 0 | 0 | 0 | 0 | 0 | 0 | 0 | 0 | 0 | 0 | 0 | 0 | 0 | 0 | 0 |
| *Flavocetraria cucullata* (Bellardi) Karnefelt & Thell | Flavcucu | 0 | 25 | 0 | 15 | 25 | 25 | 25 | 0 | 25 | 15 | 25 | 25 | 0 | 0 | 23 | 25 | 0 | 24 | 6 | 5 |
| *Nephroma expallidum (*Nyl.) Nyl. | Nephexpa | 0 | 0 | 0 | 0 | 0 | 0 | 0 | 0 | 0 | 0 | 0 | 0 | 0 | 0 | 0 | 0 | 0 | 0 | 0 | 0 |
| *Ochrolechia inaequatula* (Nyl.) Zahlbr. | Ochrinae | 0 | 0 | 0 | 0 | 0 | 0 | 0 | 0 | 0 | 0 | 0 | 0 | 0 | 0 | 0 | 0 | 0 | 0 | 0 | 0 |
| *Peltigera aphtosa* (L.) Willd. | Peltapht | 0 | 2 | 0 | 0 | 0 | 0 | 0 | 0 | 0 | 8 | 0 | 0 | 0 | 0 | 0 | 4 | 1 | 0 | 10 | 1 |
| *Peltigera frippii* Holt.-Hartw. | Peltfrip | 0 | 0 | 0 | 0 | 0 | 0 | 0 | 0 | 0 | 0 | 0 | 0 | 0 | 0 | 0 | 0 | 0 | 0 | 0 | 0 |
| *Peltigera leucophlebia* (Nyl.) Gyelnik | Peltleuc | 0 | 0 | 0 | 0 | 0 | 0 | 0 | 0 | 0 | 0 | 0 | 0 | 0 | 0 | 0 | 0 | 0 | 0 | 0 | 0 |
| *Peltigera occidentalis* (Å.E. Dahl) Kristinsson | Peltocci | 0 | 0 | 0 | 0 | 0 | 0 | 0 | 0 | 0 | 0 | 0 | 1 | 0 | 0 | 0 | 0 | 0 | 0 | 0 | 0 |
| *Peltigera scabrosa* Th. Fr. | Peltscab | 0 | 0 | 0 | 11 | 0 | 0 | 0 | 0 | 0 | 0 | 0 | 0 | 0 | 0 | 0 | 0 | 0 | 0 | 0 | 0 |
| *Protopannaria pezizoides* (Weber) P.M.Joerg.& S.Ekman | Protpezi | 0 | 0 | 0 | 0 | 0 | 0 | 0 | 0 | 0 | 1 | 0 | 0 | 0 | 0 | 0 | 0 | 0 | 0 | 0 | 0 |
| *Stereocaulon alpinum* Laurer ex Funck | Steralpi | 0 | 0 | 0 | 0 | 0 | 0 | 0 | 0 | 0 | 0 | 0 | 0 | 1 | 0 | 0 | 0 | 0 | 0 | 0 | 0 |
| *Thamnolia vermicularis* (Sw.) Ach. ex Schaerer | Thamverm | 0 | 0 | 0 | 0 | 0 | 0 | 0 | 0 | 0 | 0 | 0 | 0 | 0 | 0 | 0 | 0 | 0 | 0 | 0 | 3 |
|  |  |  |  |  |  |  |  |  |  |  |  |  |  |  |  |  |  |  |  |  |  |
